# Shifts in Demographics and Characteristics Among Patients Leaving Against Medical Advice From Before, During, and After the COVID-19 Pandemic

**DOI:** 10.7759/cureus.90290

**Published:** 2025-08-17

**Authors:** Aaron Shaykevich, David Yagudayev, Jamie Romeiser, Ivayla Geneva

**Affiliations:** 1 Public Health and Preventative Medicine, State University of New York Upstate Medical University, Syracuse, USA; 2 Infectious Diseases, State University of New York Upstate Medical University, Syracuse, USA

**Keywords:** ama discharge, covid-19, discharge against medical advice (ama), medical mistrust, social deprivation, social determinants

## Abstract

Introduction: Discharges against medical advice (AMA) present a significant public health challenge and are associated with increased readmission and mortality rates. The COVID-19 pandemic altered the healthcare landscape regarding public trust in the United States, which may have impacted the demographic and clinical profile of patients with AMA discharges. The aim of this study is to identify potential changes in the demographics of AMA discharges before, during, and after the COVID-19 pandemic at a large academic medical center.

Methods: This non-interventional retrospective cohort study included adult inpatients (n = 1,560) discharged AMA during three time periods: pre-COVID-19, during COVID-19, and post COVID-19. Demographic variables, the Social Deprivation Index (SDI), and comorbidities were analyzed via bivariate analysis and subsequently via multinomial logistic regression.

Results: During COVID-19, Black patients made up a larger portion of AMA discharges compared to before the pandemic. The length of hospital stay before leaving AMA increased during and after the pandemic (43.1% discharged within two days before COVID-19, compared to 40.7% during the pandemic and 28.3% after the pandemic). In the post-COVID-19 period, AMA discharges were associated with higher SDI scores (M = 72.05 (SD = 28.67)) compared to before and during COVID-19 (M = 68.03 (SD = 29.43) before, 67.71 (SD = 29.87) during). Patients were also more likely to stay longer before leaving AMA than during COVID-19. Additionally, the frequency of AMA discharges originating from the ICU increased significantly following the resolution of the pandemic.

Conclusion: The determinants of AMA discharges have a temporal association with the COVID-19 pandemic. Findings suggest that patients leaving AMA are now more likely to be socially disadvantaged, from minority backgrounds, to have longer hospital stays, and to be leaving directly from the ICU. Targeted interventions to improve patient trust and support in these populations are needed to reduce AMA discharges and improve outcomes.

## Introduction

A discharge against medical advice (AMA) happens when a patient chooses on their own accord to leave a hospital or emergency department before their provider recommends discharge [1]. AMA discharges are a public health problem, as they have been associated with an increased risk for adverse patient outcomes. Specifically, this patient population has been documented to have a higher readmission rate and, notably, a higher mortality rate [2, 3].

While patients in the United States have the legal right to decline recommended care and hospital stays, AMA discharges pose great safety and systemic challenges. AMA discharges comprise approximately 1% to 2% of United States hospital admissions and have even higher rates in the emergency department setting [1]. Longitudinal data from the United States Nationwide Inpatient Sample has shown a 1.9% average annual increase in AMA discharges from 2002 to 2011 [4], and many studies have found similar trends continue more recently [5, 6].

There are many social determinants and risk factors linked to AMA discharge. These include social deprivation status, age, sex, race, insurance type and coverage, and substance use [7-9]. There are also psychological, social, and financial pressures that play a role and heighten AMA discharge risk [10]. Patients cite diverse motivators for AMA discharge, which include refusal of procedures, wait time dissatisfaction, financial burdens, caregiving responsibilities, and others [11].

The breakdown in trust and communication is believed to be a key player in AMA discharges. Patients who feel unheard and/or disrespected are more likely to leave prematurely [12]. Recently, the COVID-19 pandemic led to a sharp decline in physician and hospital trust in the United States. In a national survey, public trust drastically fell from 71.5% in 2020 to 40.1% in 2024 [13]. This significant decline in public trust was held across demographics and correlated with lower COVID-19 and influenza vaccine uptake.

Our research question is on how the demographics of patients leaving AMA changed before, during, and after the COVID-19 pandemic. This study should provide a greater understanding of the demographic shifts of AMA patients, which can help identify emerging at-risk groups and guide interventions to reduce preventable discharges and improve patient outcomes. 

## Materials and methods

This retrospective cohort study aimed to identify the changes in the demographic profile of patients leaving via AMA discharges at the State University of New York (SUNY) Upstate Medical University Hospital, Syracuse, NY. This study utilized three years of patient visits: one year prior to COVID-19 (January 1, 2019 to December 31, 2019), one year during the early COVID-19 pandemic (March 1, 2020, to February 28, 2021), and one year after the COVID-19 pandemic (May 11, 2023 to May 10, 2024). Using equal one-year intervals for each period allowed for direct comparison between phases while minimizing variability within a timeframe. The study population included all patients aged 18 years and older admitted to the hospital during this time frame that were discharged AMA. All data used in this study were managed in accordance with relevant ethical guidelines and institutional policies. This study was reviewed by the SUNY Upstate Institutional Review Board and determined to be exempt from IRB review under Exemption Category 4(iii) in accordance with 45 CFR 46.104(d). A waiver of the Health Insurance Portability and Accountability Act (HIPAA) authorization was granted.

Patient demographic and visit data were collected from the EPIC electronic health record system (Epic Systems Corporation, Verona, WI) using EPIC’s data extraction tool SlicerDicer. All statistical analyses were performed using IBM SPSS Statistics software, version 29.0 (IBM Corp., Armonk, NY), with statistical significance assessed at the 0.05 level.

Independent variables included patients’ demographics such as age, sex, and race. Patients’ zip codes were used to determine the patients’ Social Deprivation Index (SDI) using the ZIP Code Tabulation Areas in the 2017-2022 dataset from the Robert Graham Center [14, 15]. The SDI is a composite measure of area-level disadvantage based on income, education, employment, housing, and other social determinants, with scores ranging from 0 to 100, with higher scores indicating greater social deprivation. Additional independent variables include comorbidities such as mental health disorders (the International Classification of Diseases, Tenth Revision, Clinical Modification (ICD-10) [16] psychiatric diagnoses such as anxiety disorders, mood disorders, personality disorders, eating disorders, post-traumatic stress disorder, psychotic disorders, intellectual disabilities, and dementia-related conditions), substance abuse (all ICD-10 substance use diagnoses except alcohol abuse), alcohol abuse, and the length of stay for each hospitalization (divided into categories: less than two days, two to eight days, eight to 25 days, more than 25 days). We also recorded whether a patient left AMA more than once in the same year.

The only missing data was for patient SDI, due to no reported zip code, and therefore, 14.6% of cases were excluded from the multivariate analysis. In order to prevent possible bias in the analysis, patients who left AMA multiple times in one year only had the first AMA included in the analysis. 

For the bivariate analysis, the chi-square test was used for categorical data, and one-way ANOVA was used for continuous variables. The variables from the bivariate analyses with p < 0.2 were then included in a multinomial logistic regression model, with the time period of “during COVID-19” as the reference level. The p-value threshold was chosen for inclusion in the multinomial logistic regression to avoid prematurely excluding variables that may not reach statistical significance in bivariate analysis but could still be important predictors in the multivariable model, consistent with established variable selection practices in epidemiologic research. A subsequent supplemental analysis was done by limiting the sample to patients who left AMA more than once within the same year.

## Results

A total of 1,560 patients were included in this study across three different periods: before COVID-19 (n = 427), during the COVID-19 pandemic (n = 514), and after COVID-19 (n = 619). Before COVID-19, the rate of AMA was 1.05%; during COVID-19, the rate increased to 1.41%; and after COVID-19, the rate of AMA increased further to 1.71%. Table 1 highlights the demographic data and patient characteristics for these three different time periods. 

**Table 1:** Demographic Data of the Study Group ^a ^Missing 14.6% of cases; AMA: against medical advice

Variables	Before COVID-19 (n = 427)	During COVID-19 (n = 514)	Post COVID-19 (n = 619)	P-value
Male, N (%)	263 (61.6%)	330 (64.2%)	373 (60.3%)	0.391
Age (Years), Mean (SD)	50.93 (15.78)	48.81 (15.80)	49.56 (16.33)	0.126
Race, N (%)				0.079
White	322 (75.4%)	363 (70.6%)	428 (69.1%)	
Black	75 (17.6%)	121 (23.5%)	139 (22.5%)	
All other	30 (7.0%)	30 (5.8%)	52 (8.4%)	
SDI, Mean (SD)^a^	68.03 (29.43)	67.71 (29.87)	72.05 (28.67)	0.038
Any Comorbidity, N (%)	75 (17.6%)	117 (22.8%)	106 (17.1%)	0.035
Alcohol Abuse, N (%)	14 (3.3%)	23 (4.5%)	21 (3.4%)	0.539
Substance Abuse, N (%)	21 (4.9%)	47 (9.1%)	48 (7.8%)	0.045
Mental Health, N (%)	45 (10.5%)	58 (11.3%)	48 (7.8%)	0.106
Length of Stay (Days), N (%)				<0.001
<2	184 (43.1%)	209 (40.7%)	175 (28.3%)	
2 to 8	148 (34.7%)	190 (37.0%)	279 (45.1%)	
8 to 25	17 (4.0%)	45 (8.8%)	76 (12.3%)	
Over 25	3 (0.7%)	6 (1.2%)	7 (1.1%)	
No Value (No Overnight Stay)	75 (17.6%)	64 (12.5%)	82 (13.2%)	
Additional AMA Discharges, N (%)	49 (11.5%)	61 (11.9%)	66 (10.7%)	0.807
Department, N (%)				0.023
ICU	48 (11.2%)	59 (11.5%)	91 (14.7%)	
Medical-Surgical Unit	348 (81.5%)	423 (82.3%)	507 (81.9%)	
Other	31 (7.3%)	32 (6.2%)	21 (3.4%)	

Most notably, the mean SDI of AMA discharges significantly changed over time, with an increase from 68.03 before COVID-19 and 67.71 during COVID-19 to 72.05 after COVID-19 (p = 0.038). Additionally, the proportion of AMA participants with a documented comorbidity changed from 17.6% before COVID-19 to 22.8% during COVID-19 and back down to 17.1% after (p = 0.035). Among the comorbidity subgroups, the only significant change was in substance abuse (p = 0.045). While not statistically significant, there appears to be a change in the proportion of AMA discharges who were Black patients, which increased from 17.6% before COVID-19 to 23.5% during and 22.5% after (p = 0.079).

Patients stayed in the hospital for longer periods post COVID-19 (p < 0.001). Short hospital stays (< two days) significantly decreased from 43.1% of AMA discharges before COVID-19 to 40.7% during and 28.3% after, while stays of two to eight days increased from 34.7% to 37.9% during and 45.1% after in the same period. The proportion of ICU admissions increased from 11.2% before COVID-19 to 11.5% during and 14.7% after, while other departments saw a decline in the number of AMA discharges from 7.3% before COVID-19 to 6.2% during and 3.4% after (p = 0.023).

The results of our regression analyses are detailed in Table 2. Comparing before COVID-19 to during, Black patients made up a significantly smaller proportion of those leaving AMA before COVID-19 than during (adjusted odds ratio (aOR): 0.665, 95% CI: 0.460-0.961). Additionally, the overall length of stay increased, and it was significantly less likely that patients would have a length of stay from eight to 25 days before leaving AMA before COVID-19 than during (aOR: 0.384, 95% CI: 0.192-0.770).

**Table 2:** Regression Analysis ^a ^Missing 14.6% of cases; SDI: Social Deprivation Index; Ref: reference group

Variables	Unadjusted (n = 1,560)			Adjusted (n = 1,332)		
	Odds Ratio	95% Confidence Interval	P-value	Odds Ratio	95% Confidence Interval	P-value
Before COVID-19						
Age	1.008	1.000, 1.016	0.044	1.009	0.999, 1.018	0.067
SDI^ a^	1.000	0.996,1.005	0.883	1.003	0.998, 1.008	0.287
No Comorbidity	1.383	1.001, 1.911	0.049	1.274	0.883, 1.837	0.195
Race						
Black	0.699	0.505, 0.967	0.031	0.665	0.460, 0.961	0.030
Other	1.127	0.665, 1.911	0.656	0.932	0.518, 1.677	0.814
White	Ref	--	--	Ref	--	--
Length of Stay (Days)						
No Value (No Overnight Stay)	1.331	0.903, 1.962	0.148	1.466	0.965, 2.228	0.073
Over 25	0.568	0.140, 2.303	0.428	0.592	0.111, 3.147	0.538
8 to 25	0.429	0.237, 0.776	0.005	0.384	0.192, 0.770	0.007
2 to 8	0.885	0.661, 1.185	0.412	0.872	0.631, 1.206	0.409
<2	Ref	--	--	Ref	--	--
Department						
ICU	0.989	0.659, 1.485	0.957	1.218	0.771, 1.923	0.397
Other	1.178	0.704, 1.969	0.533	1.537	0.856, 2.760	0.150
Medical-Surgical Unit	Ref	--	--	Ref	--	--
After COVID-19						
Age	1.003	0.996, 1.010	0.428	1.000	0.991, 1.008	0.915
SDI^ a^	1.005	1.001, 1.009	0.020	1.005	1.001, 1.010	0.026
No Comorbidity	1.426	1.063, 1.913	0.018	1.261	0.911, 1.746	0.162
Race						
Black	0.974	0.736, 1.290	0.856	0.921	0.671, 1.264	0.609
Other	1.47	0.918, 2.354	0.109	1.221	0.727, 2.050	0.45
White	Ref	--	--	Ref	--	--
Length of Stay (Days)						
No value (No overnight stay)	1.53	1.043, 2.246	0.030	1.679	1.115, 2.529	0.013
Over 25	1.393	0.460, 4.223	0.558	1.094	0.284, 4.21	0.896
8 to 25	2.017	1.326, 3.069	0.001	2.221	1.388, 3.555	<.001
2 to 8	1.754	1.335, 2.303	<.001	1.827	1.357, 2.460	<.001
<2	Ref	--	--	Ref	--	--
Department						
ICU	1.287	0.905, 1.830	0.160	1.554	1.039, 2.324	0.032
Other	0.548	0.311, 0.964	0.037	0.751	0.407, 1.387	0.360
Medical-Surgical Unit	Ref	--	--	Ref	--	--

Comparing after COVID-19 to during, higher SDI scores were associated with leaving AMA after COVID-19, meaning that SDI significantly increased post COVID-19 compared to during (aOR: 1.005, 95% CI: 1.001-1.010). Additionally, the length of stays before leaving AMA increased largely, and patients were more likely to stay two to eight days (aOR: 2.221, 95% CI: 1.388-3.555) and eight to 25 days (aOR: 1.827, 95% CI: 1.357-2.460) before leaving AMA after COVID-19 compared to during. AMA patients were also more likely to have left the ICU after COVID-19 than during (aOR: 1.554, 95% CI: 1.039-2.324).

A supplementary analysis, shown in Table 3, was done exclusively on patients who left AMA more than once within the same year. There were 176 patients who left AMA several times, but no statistically significant differences were found across the three time periods. However, it should be noted that these patients had noticeably higher rates of comorbidities compared to the general cohort who left AMA once, with 17.6% compared to 38.8% before COVID-19, 22.8% compared to 42.6% during COVID-19, and 17.1% compared to 47.0% after COVID-19.

**Table 3:** Demographic Data of Participants Who Left AMA Multiple Times ^a ^Missing 8.0% of cases; AMA: against medical advice

Variables	Before COVID-19 (n = 49)	During COVID-19 (n = 61)	Post COVID-19 (n = 66)	P-value
Male, N (%)	33 (67.3%)	33 (54.1%)	46 (69.7%)	0.154
Age, Mean (SD)	50.00 (14.51)	46.28 (15.71)	44.62 (13.97)	0.151
Race, N (%)				0.905
White	34 (69.4%)	41 (67.2%)	45 (68.2%)	
Black	12 (24.5%)	18 (29.5%)	19 (28.8%)	
All Other	3 (6.1%)	2 (3.3%)	2 (3.0%)	
SDI, Mean (SD)^a^	72.57 (25.17)	73.29 (27.97)	74.70 (31.84)	0.928
Any comorbidity, N (%)	19 (38.8%)	26 (42.6%)	31 (47.0%)	0.677
Alcohol Abuse, N (%)	4 (8.2%)	3 (4.9%)	9 (13.6%)	0.221
Substance Abuse, N (%)	4 (8.2%)	13 (21.3%)	16 (24.2%)	0.075
Mental Health, N (%)	14 (28.6%)	12 (19.7%)	13 (19.7%)	0.445

## Discussion

In this large study of 1,560 hospitalized patients who left AMA, meaningful shifts in the characteristics related to AMA discharges can be observed before and after COVID-19, compared to during the pandemic. These data and shifts provide important insights and significant clinical and policy implications. 

Patient demographics changed significantly over time. Black patients made up a larger proportion of AMA patients during and after COVID-19 compared to before COVID-19. This suggests that Black patients may be leaving AMA at higher rates than prior to the pandemic and additionally highlights that more effort into interventions and programs targeting historically marginalized communities may be beneficial. The current literature indicates that Black patients have greater odds of generally leaving AMA even after adjusting for various confounders such as age, sex, income, severity of illness, and others [9, 17, 18]. Our findings suggest that this issue persisted and may have worsened during and post COVID-19.

Our study also found that compared to before and during the pandemic, the social deprivation of those leaving AMA increased after COVID-19. Social deprivation has been correlated with worse outcomes and increased need for healthcare due to COVID-19 [19, 20], which, together with our findings, suggests that socially disadvantaged individuals may have an increased need for care after COVID-19, of which some may leave AMA. While there is certainly a need for additional research on this, there are relevant policy implications with this finding. Importantly, as seen by the elevated SDI after COVID-19 among AMA patients, interventions aiming to prevent AMA discharges would benefit from targeting communities with increased social deprivation.

Prior to COVID-19, longer stays of eight to 25 days appeared relatively infrequently in AMA discharges. However, after COVID-19, a major shift occurred, and longer stays became more frequent among those with AMA discharges. This ultimately means that more hospital resources are being used for patients who do not finish the full course of treatment. This may be seen as a success, since patients are more likely to get at least minimal care and partial treatment. However, it also means that resources are inefficiently utilized and diverted away from those who would receive the full benefit of a full course of treatment. These results should prompt further research into whether there are differences in outcomes among those leaving AMA based on how long they remained in the hospital before they were discharged. 

Although no statistically significant findings were found in the recurrent AMA subgroup, the subgroup consistently showed higher rates of comorbidities. This is in line with earlier studies in both emergency department and inpatient settings, which reported that patients with psychiatric illness, substance use disorders, and prior history of AMA were among the strongest predictors for future AMA discharges [21-23].

Overall, our findings suggest that in the novel healthcare landscape after COVID-19, it is crucial to redefine the typical profile of patients leaving AMA and to identify the best strategies for combating and mitigating the number of AMA discharges. After COVID-19, interventions should put additional emphasis on communicating with groups with elevated SDI. Vaccine hesitancy has also risen notably in the wake of the COVID-19 pandemic, driven in part by declining trust in healthcare systems [13]. This erosion of trust, well documented for vaccinations, may similarly underlie/influence decisions to leave hospitals AMA. The same communication strategies effective in mitigating vaccine hesitancy may very well be helpful in decreasing AMA discharge rates. Just as declining trust has fueled vaccine refusal, it may also be an important factor that pushes patients toward AMA discharge. Further research into the connection between AMA discharges and larger medical utilization (such as vaccine uptake) would be beneficial. For example, a recent study found that flu vaccine uptake was associated with cancer screening [24]. This can guide co-targeted interventions that promote a larger overall trust and willingness to utilize medical systems. Additionally, hospitals and health systems looking to prevent AMA among patients should consider the ICU as an area of increased risk of AMA, as well as patients with longer lengths of stay. 

There are some limitations with our study. Firstly, there were limitations regarding the acquisition of patient zip codes, and as such, this variable featured the most missing data. Despite this, the SDI variable was statistically significant. Secondly, it would have been beneficial to separate medical and surgical departments. However, this was not possible using the extraction tool SlicerDicer. Lastly, there are likely additional variables that have changed among AMA discharges over time. However, we chose the variables that are most established in the literature and in the authors’ clinical experience.

To summarize, the demographic and clinical distribution of AMA discharges has changed significantly in many ways from before COVID-19. Among demographics, the percentage of AMA patients who are Black and the mean SDI have increased from before COVID-19. There was an increase in patients leaving AMA with comorbidities during COVID-19, but it returned to baseline levels after the pandemic. Among clinical variables, the overall length of stay has increased for AMA patients, and a higher proportion are from the ICU. 

## Conclusions

In conclusion, this study demonstrates that the demographic and clinical profile of patients being discharged AMA has shifted significantly in association with the COVID-19 pandemic. Patients leaving AMA after the pandemic were more likely to be Black patients, come from areas of greater social deprivation, have longer hospital stays, and leave directly from the ICU. These findings emphasize the evolving nature of healthcare challenges and patient behavior post COVID-19 and highlight the urgent need for targeted and equitable interventions. Hospitals should implement novel retention strategies, particularly in high-acuity settings like the ICU. Additionally, healthcare systems would benefit from communication strategies focusing on trust in healthcare systems tailored to those in areas of high social deprivation. Lastly, future research should assess the larger effects of COVID-19 on healthcare trust and the evolving healthcare landscape.
